# LncRNA RP11-79H23.3 Functions as a Competing Endogenous RNA to Regulate PTEN Expression through Sponging hsa-miR-107 in the Development of Bladder Cancer

**DOI:** 10.3390/ijms19092531

**Published:** 2018-08-26

**Authors:** Hong Chi, Rui Yang, Xiaying Zheng, Luyu Zhang, Rong Jiang, Junxia Chen

**Affiliations:** 1 Department of Cell Biology and Genetics, Chongqing Medical University, Chongqing 400016, China; 15310432691@163.com (H.C.); zbyr1224@163.com (R.Y.); pocahontas7@163.com (X.Z.); 2 Molecular Medicine and Cancer Research Center, Chongqing Medical University, Chongqing 400016, China; 13983122331@163.com; 3 Laboratory of Stem Cells and Tissue Engineering, Chongqing Medical University, Chongqing 400016, China; 13150535015@163.com

**Keywords:** lncRNA, ceRNA, miR-107, bladder cancer, PTEN

## Abstract

Accumulating evidence indicates that the aberrant expression of long noncoding RNAs (lncRNAs) is involved in tumorigenesis and cancer development. However, the biological functions and underlying mechanisms of lncRNAs in bladder cancer (BC) remain largely unknown. Here, we analyzed the lncRNA and mRNA expression profiles in BC using a microarray assay. We found that lncRNA RP11-79H23.3 and phosphatase and tensin homolog (PTEN) were significantly downregulated in BC tissues and cells. Meanwhile, RP11-79H23.3 expression was negatively correlated with clinical stage in BC. Functionally, we found that overexpression of RP11-79H23.3 could suppress cell proliferation, migration, and cell cycle progression, rearrange the cytoskeleton, and induce apoptosis in vitro. Moreover, upregulation of RP11-79H23.3 inhibited the angiogenesis, tumorigenesis, and lung metastasis in vivo, whereas RP11-79H23.3 knockdown exerted a contrary role. Mechanistically, we identified that RP11-79H23.3 could directly bind to miR-107 and abolish the suppressive effect on target gene PTEN, which leads to inactivation of the PI3K/Akt signaling pathway. Taken together, we first demonstrated that RP11-79H23.3 might suppress the pathogenesis and development of BC by acting as a sponge for miR-107 to increase PTEN expression. Our research revealed that RP11-79H23.3 could be a potential target for diagnosis and therapy of BC.

## 1. Introduction

Bladder cancer (BC) is a common cancer worldwide, with an estimated 429,800 new cases diagnosed and 165,100 deaths occurring annually [1]. Bladder cancer patients can be treated by radiation, surgery, chemotherapy, and other methods of treatment to improve the survival rate of patients. However, BC has a poor prognosis and a high recurrence rate after surgery, with the 5-year overall survival rate being only 50–60% [2,3]. Therefore, discovering new molecular biomarkers and the molecular mechanism underlying the development and progression of BC are desperately needed.

Long noncoding RNAs (lncRNAs) are a class of RNA transcripts greater than 200 nt in length without the function of protein encoding [4]. Although only a small number of functional lncRNAs have been well characterized to date, they have been shown to regulate gene expression at various levels, including chromatin modification, transcription, and post-transcriptional processing [5]. Increasing evidence suggests that lncRNAs play essential roles in tumor development and could potentially serve as prognostic markers and therapeutic targets [6,7,8]. We found that a lncRNA-RP11-79H23.3 (RP11-79H23.3, RP11-79H), with a length of 2994 nucleotides from chromosome 8q21.13, was significantly downregulated in bladder cancer using microarray analysis. Interestingly, phosphatase and tensin homolog (PTEN) was also downregulated concomitantly with lncRNA-RP11-79H23.3 expression and the expression of RP11-79H23.3 was negatively correlated with the clinical stage of BC. However, the biological significance and exact molecular mechanism are still unknown.

MicroRNAs (miRNAs) are a class of highly conserved small noncoding RNAs with a length of 18–25 nucleotides that could inhibit protein translation or degrade target mRNAs to regulate gene expression at a post-transcriptional level [9]. It is well known that miRNAs can act as either an oncogene or tumor suppressor in the development and progression of cancer [10]. Recently, a new regulatory mechanism has been proposed in which RNA transcripts can crosstalk with each other by competing for shared miRNA response elements (MREs). In this case, lncRNAs may function as competing endogenous RNAs (ceRNAs) to sponge miRNAs, thereby modulating the depression of miRNA targets and imposing an additional level of post-transcriptional regulation [11]. Theoretically, any RNA transcript with MREs might act as a ceRNA. If the balance of the ceRNA intricate network is disturbed, it could lead to cancer. Studies have demonstrated that a dysregulated ceRNA–ceRNA interaction network could contribute to the initialization and development of a variety of cancers, including hepatocellular carcinoma, gastric cancer, colorectal cancer, and bladder cancer [12,13,14,15]. However, the molecular mechanisms and the interaction between RP11-79H23.3 and miR-107 in BC are still unclear.

PTEN is a ubiquitous tumor suppressor that plays a critical role in cell growth and proliferation, and the functional inactivation of PTEN is associated with the pathogenesis and development of many solid tumors [16]. PTEN is a negative regulator of the PI3K/Akt signaling pathway. Calderaro et al. found that PI3K/AKT pathway activation occurred in the entire spectrum of BC regardless of stage in 129 bladder cancer samples [17]. In addition, loss of PTEN function contributes to hyperactivation of the PI3K/AKT pathway, which could accelerate cell growth, invasion, and metastasis in BC [18]. A previous report demonstrated that 72% of 131 muscle-invasive bladder cancer specimens revealed dysregulation of the PI3K/Akt signaling pathway [19]. According to target prediction using microRNA.org, the 3′-untranslated region (UTR) of PTEN is complementary to the seed region of miR-107. However, whether miR-107 can target PTEN to regulate the PI3K/AKT signaling pathway in BC has not been reported so far.

Here, we have identified a lncRNA termed lncRNA-RP11-79H23.3, the expression of which is significantly downregulated in BC tissues and cells, and the expression of RP11-79H23.3 is negatively correlated with clinical stage of BC. The present study was designed to determine whether and how RNA-RP11-79H23.3 regulates the expression of PTEN in the carcinogenesis and progression of BC. Function assays demonstrated that overexpression of RP11-79H23.3 could inhibit BC cell growth, invasion, and induce apoptosis, implying a possible role as tumor suppressor lncRNA in BC. Mechanistically, we found that RP11-79H23.3 could function as a ceRNA to regulate the target gene PTEN by sponging miR-107, which is involved in the progression of BC. Our results are of biological and clinical significance for the diagnosis and therapy of BC.

## 2. Results

### 2.1. RP11-79H23.3 Is Downregulated in BC Tissues and Cells and Negatively Correlated with miR-107 Expression

**Figure 1:**
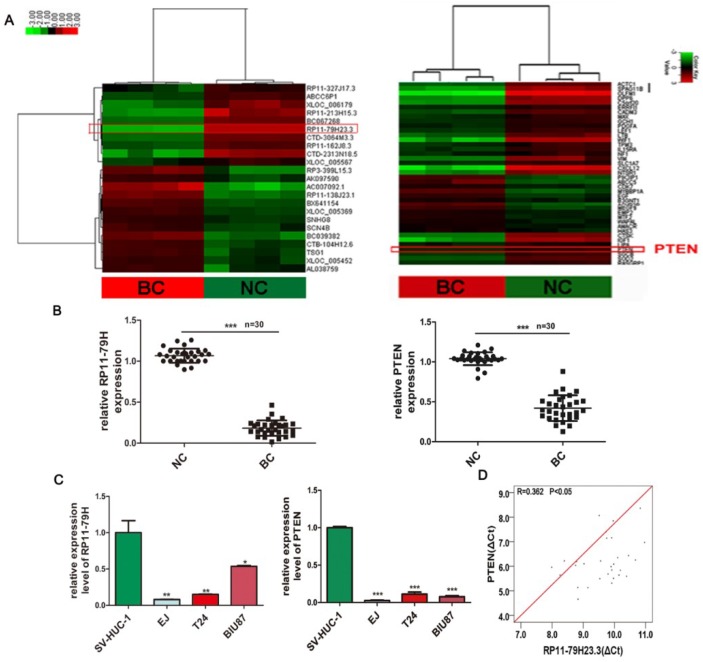
The expression of RP11-79H23.3 and phosphatase and tensin homolog (PTEN) in bladder cancer (BC) tissues and cells and the relationship between them. (**A**) Heat maps showed that the profiles of differentially expressed long noncoding RNAs (lncRNAs) (**left**) and mRNA (**right**) in bladder carcinoma tissues and adjacent noncarcinoma tissues (*n* = 4) using microarray with fold change ≥2 and *p*-value < 0.05. Each row represents the expression level of a lncRNA/mRNA and each column represents an individual tissue sample. Red represents high expression levels and green represents low expression levels. (**B**) RP11-79H23.3 and PTEN expressions were evaluated by RT–qPCR in 30 cases of bladder cancer tissues and adjacent normal tissues. (**C**) The expressions of RP11-79H23.3/PTEN were measured in three BC cell lines and one normal bladder cell line. (**D**) A positive correlation between RP11-79H23.3 expression and PTEN level was determined by Pearson analysis in BC. Data is shown as mean ± SD (standard deviation). * *p* < 0.05; ** *p* < 0.01; *** *p* < 0.001.

To explore the biological functions of lncRNAs in BC, microarray analysis of lncRNA and mRNA expression profiles was performed as previously reported [15]. Compared with paracancerous normal tissues, we found that 6137 lncRNAs and 4416 mRNAs were differentially expressed, including 3217 upregulated and 2920 downregulated lncRNAs as well as 2472 upregulated and 1944 downregulated mRNAs, with a fold change ≥2.0, *p* <0.05, and FDR (false discovery rate) < 0.05 in four bladder cancer tissues (Figure 1A). Among these, lnRNA RP11-79H23.3 was one of the most significantly downregulated lncRNAs and PTEN was one of the most markedly downregulated mRNAs. The qRT-PCR (Quantitative real time polymerase chain reaction) assays showed that RP11-79H23.3 and PTEN expressions were significantly downregulated in BC tissues compared with adjacent normal tissues from 30 patients (Figure 1B). Interestingly, the RP11-79H23.3 expression was negatively correlated with the tumor–node–metastasis (TNM) stage. Relationships between RP11-79H23.3 expression and clinical characteristics of the BC patients are shown in Table 1. Next, the expressions of RP11-79H23.3 and PTEN were further determined in bladder cancer cell lines EJ, T24, and BIU87 and the normal bladder cell line SV-HUC-1 by qRT-PCR. The data also showed that the levels of RP11-79H23.3 were significantly downregulated in three kinds of BC cells. Moreover, PTEN expressions were remarkably downregulated in BC cells compared with normal bladder epithelial cells (Figure 1C). Pearson correlation analysis revealed that the expression of RP11-79H23.3 was positively correlated with the level of PTEN in BC, *r* = −0.641 (Figure 1D). The data suggest that the correlation between expression of RP11-79H23.3 and PTEN might be involved in tumorigenesis and development of BC.

### 2.2. RP11-79H23.3 Modulates BC (Bladder Cancer) Cell Proliferation, Migration, and Invasion

**Figure 2:**
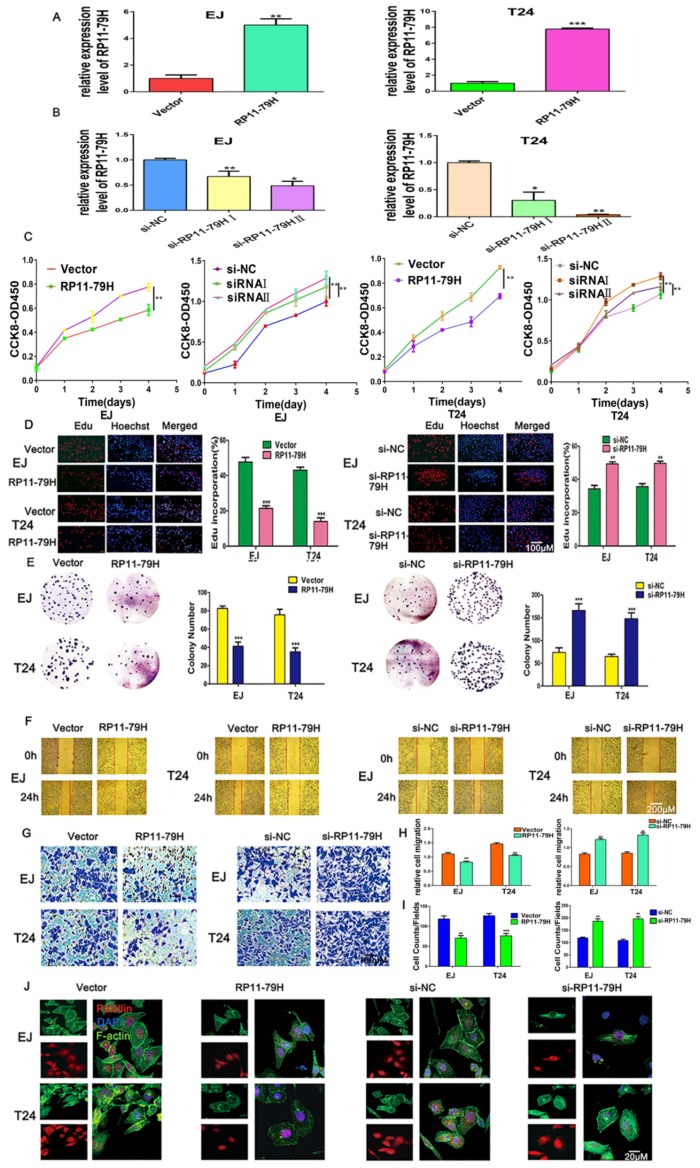
RP11-79H23.3 influences BC cell proliferation, migration, and invasion in vitro. (**A**,**B**) The expressions of RP11-79H23.3 was detected in BC cells transfected with pIRES2-RP11-79H23.3 or si-RNA fragments (si-RP11-79H23.3I, si-RP11-79H23.3II) by qRT-PCR. (**C**–**E**) The cell proliferation ability was assessed by CCK-8, EdU, and colony formation assays after upregulation and downregulation of RP11-79H23.3 in EJ and T24 cells. CCK-8 cell growth curve, relative quantification of EdU-incorporating cells, and soft agar colony formation numbers are shown, respectively (scale bar, 100 μm). (**F**,**G**) The cell migration and invasion capacities were detected in upregulating or downregulating RP11-79H23.3 cells by wound healing and transwell assays (scale bar, 200 μm; scale bar, 100 μm. (**H**,**I**) Quantitative analyses of wound healing and transwell assay are shown. Each experiment was independently repeated at least three times. Data is shown as mean ± SD. (**J**) Cytoskeleton was surveyed by phalloidine-FITC staining of microfilament and immunofluorescence staining for paxillion in BC cells under confocal laser scanning microscopy when RP11-79H23.3 was upregulated or downregulated (scale bar, 20 μm). * *p* < 0.05; ** *p* < 0.01; *** *p* < 0.001.

The expression of RP11-79H23.3 was examined in RP11-79H23.3 overexpression and RP11-79H23.3 knockdown BC cells by qRT-PCR. The result showed that the levels of RP11-79H23.3 were significantly upregulated in BC cells transfected with pIRES2-RP11-79H23.3. Conversely, the expressions of RP11-79H23.3 were remarkably decreased in BC cells transfected with si-RNA fragments (si-RP11-79H23.3I and si-RP11-79H23.3II) (Figure 2A,B). To investigate the functions of RP11-79H23.3, the effects of RP11-79H23.3 on cell proliferation, migration, and invasion were explored when RP11-79H23.3 was downregulated or upregulated. The CCK-8 results showed that cell viability with transfection of the pIRES2-RP11-79H23.3 was significantly decreased compared with empty vector group (Figure 2C). EdU and colony formation assays further verified that upregulation of RP11-79H23.3 markedly inhibited the number of EdU-positive cells and colonies, while RP11-79H23.3 knockdown exhibited the opposite effects (Figure 2D,E). Wound healing and transwell assays indicated that siRP11-79H23.3 could significantly accelerate the migration and invasion of EJ and T24 cells compared with vector control groups, whereas the number of migrating and invading cells in the pIRES2-RP11-79H23.3 groups were significantly decreased compared with vector control groups (Figure 2F–I). It has been known that actin filaments are involved in adhesion and migration of tumor cells to provide support and motor activity. Cytoskeletal protein paxillin plays an important role in integrin signal transduction. Accordingly, F-actin and protein paxillin were detected with fluorescent phalloidin and immunofluorescence respectively. When RP11-79H23.3 was downregulated, more abundant actin filaments and a brighter fluorescent signal of paxillin were observed, whereas upregulation of RP11-79H23.3 significantly suppressed stress fiber formation and paxillin expression (Figure 2J).

### 2.3. Upregulation of RP11-79H23.3 Induces Apoptosis and Regulates Cell Cycle of BC Cells

**Figure 3:**
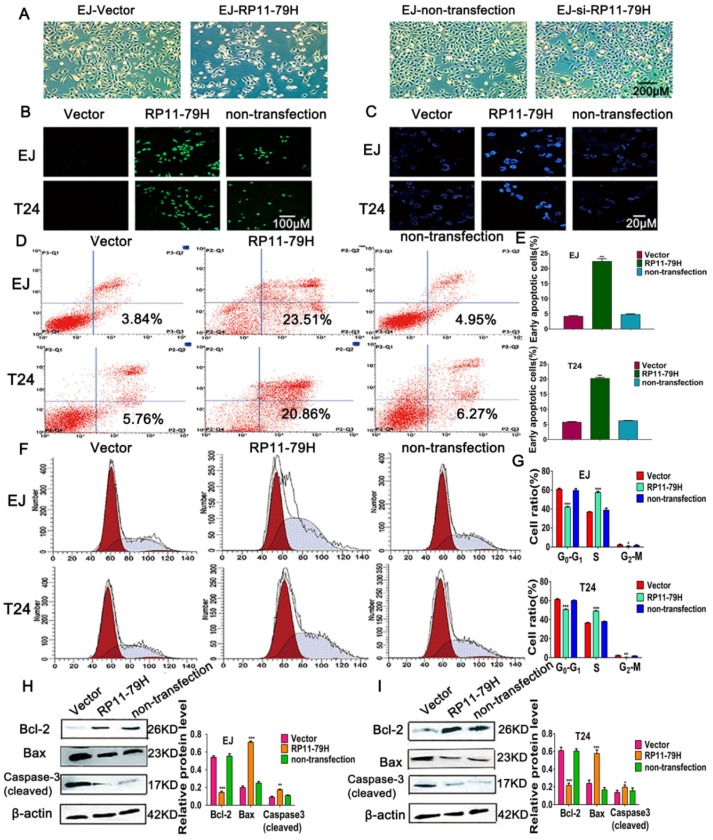
Upregulation of RP11-79H23.3 induces apoptosis and represses BC cell cycle progression. (**A**) The living cell photomicrographs were shot using a phase contrast microscope (scale bar, 200 μm). (**B**) Representative photographs of TUNEL staining of cells were observed (scale bar, 100 μm). (**C**) The typical images of Hoechst 33342 staining were taken (scale bar, 20 μm). (**D**,**E**) Apoptosis was determined by flow cytometric analysis of Annexin-V/PI staining and the apoptotic index was calculated. (**F**,**G**) Flow cytometry images by propidium iodide staining and cell cycle distribution were indicated. (**H**,**I**) The expressions of apoptosis-regulatory proteins were determined by Western blot. Each experiment was independently repeated at least three times. Data is shown as mean ± SD, * *p* < 0.05, ** *p* < 0.01, *** *p* < 0.001.

To determine whether RP11-79H23.3 functions as a tumor suppressor, cellular apoptosis was evaluated by flow cytometry, Hoechst33342 staining, TUNEL (TdT-mediated dUTP Nick-End Labeling) assays, and Western blotting after transfection with pIRES2-RP11-79H23.3. Live cell images showed that upregulation of RP11-79H23.3 suppressed the malignant phenotype, including a smaller nucleus, fewer division phases, and slower growth compared with the control group, whereas siRP11-79H23.3 group cells indicated an enhanced malignant feature, including overlapping growth and more division phases (Figure 3A). TUNEL assay revealed that upregulation of RP11-79H23.3 expression led to a significantly increased number of TUNEL-positive cells compared with control groups (Figure 3B). Meanwhile, Hoechst33342 staining found that pIRES2-RP11-79H23.3 group cells displayed typical apoptotic morphology characteristics, such as chromatin condensation, nuclear shrinkage, apoptotic body, nuclear fragmentation, and brighter fluorescent. However, the apoptotic features did not appear in control cells (Figure 3C). Furthermore, flow cytometry analysis with Annexin V/PI double staining showed that the percentages of apoptotic cells in pIRES2-RP11-79H23.3 cell groups were significantly higher than those of the control group cells, respectively (Figure 3D,E). The cell cycle analysis was performed by flow cytometry. The data showed that upregulation of RP11-79H23.3 obviously enhanced the percentage of cells in the S phase and decreased the percentage of G2-M-phase and G0/G1-phase cells compared with the controls, indicating that RP11-79H23.3 induced S-phase arrest of BC cells (Figure 3F,G). To understand the molecular basis of the apoptosis after upregulation RP11-79H23.3, the expressions of apoptosis-related proteins were detected by Western blotting. The results found the expressions of activated (cleaved) caspase-3 and proapoptotic protein Bax were markedly enhanced in pIRES2-RP11-79H23.3 group cells, while RP11-79H23.3 overexpression resulted in a decrease of Bcl-2 expression (Figure 3H,I).

### 2.4. RP11-79H23.3 Suppresses Tumorigenesis, Metastasis, and Angiogenesis of BC Cells In Vivo

**Figure 4:**
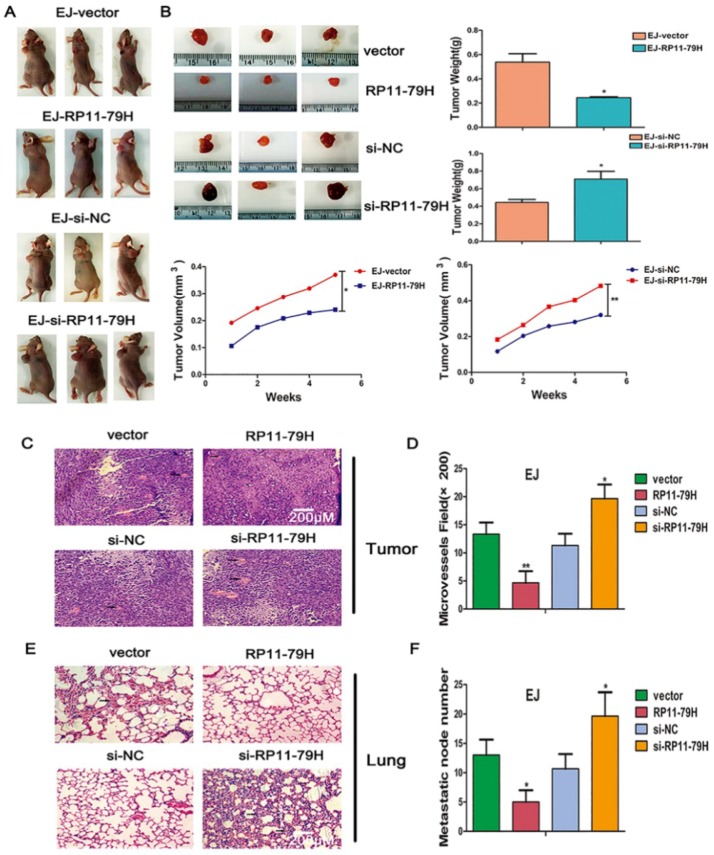

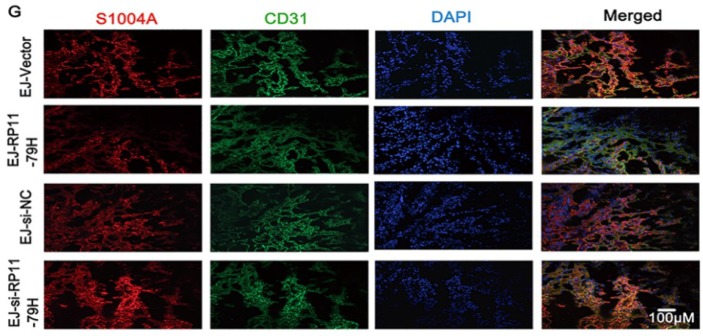
RP11-79H23.3 affects the tumor growth, spontaneous metastasis, and angiogenesis of BALB/c nude mouse xenograft model. 2.5 × 10^6^ diverse kinds of EJ cells were inoculated subcutaneously into the back of the BALB/C nude mice. After 5 weeks, the mice were sacrificed. (**A**) Representative images of the tumor-bearing BALB/C nude mice are shown. (**B**) The representative images of tumors, growth curves of xenograft tumors, and tumor weight analysis are indicated. The tumor volumes were measured every week and the data were normalized to control group, *n* = 8. (**C**) HE staining of tumors indicate microvessels among the tumors with arrows (scale bar, 200 μm). (**D**) The microvessel density analysis is shown. (**E**) HE staining of lung sections show metastasis nodules and metastatic tumor cells by arrows (scale bar, 200 μm). (**F**) The metastatic nodule number is displayed. (**G**) Immunofluorescent stainings were implemented with antibodies against CD31 and S100A4 of vascular endothelial cells (scale bar, 100 μm). Data are shown as mean ± SD, * *p* < 0.05, ** *p* < 0.01.

To further explore the biological function of RP11-79H23.3 in vivo, we established a human bladder carcinoma xenograft model in nude mice. 2.5 × 10^6^ various kinds of EJ cells were subcutaneously inoculated into the back of nude mice. After 35 days, all the mice were sacrificed. We found that RP11-79H23.3 overexpression evidently inhibited tumorigenicity of BC cells, while the siRP11-79H23.3 cell group showed a significantly higher tumor growth rate and heavier tumor weight compared with control groups (Figure 4A,B). Furthermore, upregulation of RP11-79H23.3 obviously suppressed tumor angiogenesis, whereas downregulation of RP11-79H23.3 led to more tumor microvessels (Figure 4C,D). In addition, compared with control groups, RP11-79H23.3siRNA resulted in a significant enhancement of spontaneous pulmonary metastasis with apparent visible lung metastatic nodes, while upregulation of RP11-79H23.3 markedly attenuated metastasis with fewer invasive tumor cells (Figure 4E,F). To further examine the effect of RP11-79H23.3 on angiogenesis, immunofluorescence assays for CD31 and S100A4 were implemented. CD31 is used primarily to demonstrate the presence of endothelial cells in histological tissue sections. This can help to evaluate the degree of tumor angiogenesis, which can imply a rapidly growing tumor. S100A4 is a member of the S100 calcium-binding protein family. It can support tumorigenesis by triggering angiogenesis. The results demonstrated that RP11-79H23.3 inhibited tumor angiogenesis, whereas si-RP11-79H23.3 led to a higher CD31 and S100A4 expression and more microvessels compared with the control group (Figure 4G).

### 2.5. RP11-79H23.3 Expression Correlates with PTEN and PI3K/AKT Signaling Pathway Molecules in BC

**Figure 5:**
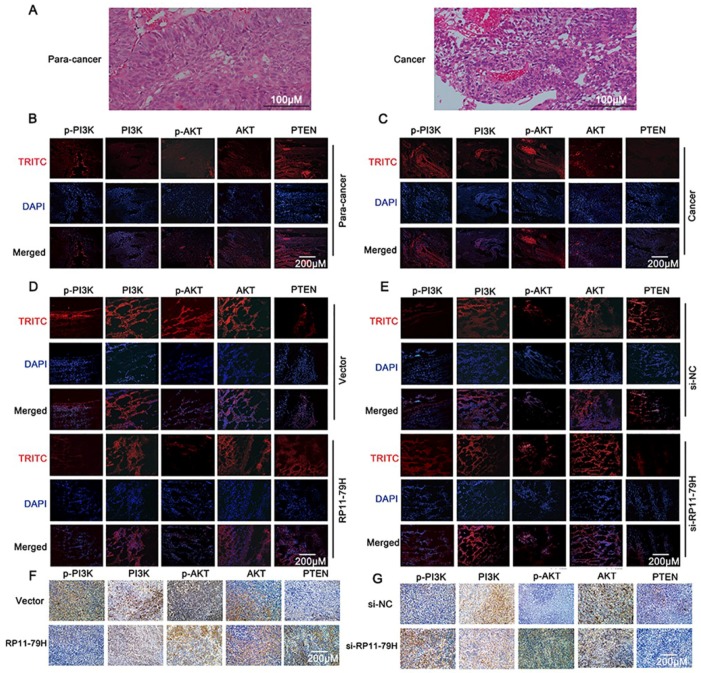
The correlation between the expression of RP11-79H23.3 and the PTEN/PI3K/AKT signaling pathway molecules was detected. (**A**) Representative images of HE-stained human bladder carcinomas and matched para-carcinomas from patients were taken (scale bar, 100 μm). (**B**,**C**) Immunofluorescence staining was performed for detection of the PTEN and PI3K/AKT signaling pathway molecules in human bladder cancer tissue and matched para-carcinomas (scale bar, 200 μm). (**D**–**G**) Immunohistochemical and immunofluorescent stainings were performed for examination of the PTEN and PI3K/AKT signaling pathway molecules in tumor tissues from nude mice after ectopic expression and knockdown of RP11-79H23.3 (scale bar, 200 μm).

To understand the underlying relationship between RP11-79H23.3 and the PTEN/PI3K/AKT signaling pathway, we performed immunofluorescence and immunohistochemistry assays in vivo. First, representative HE-stained images are shown from 30 pairs of BC tissues and matched adjacent nontumor tissues in Figure 5A. Subsequently, we determined the expressions of PTEN and PI3K/AKT signaling pathway molecules in human BC tissues from 30 patients by immunofluorescence assay. The result showed that BC tissues displayed much weaker positive staining of PTEN level as well as stronger p-PI3K and p-Akt expressions compared with adjacent nontumor tissues (Figure 5B,C). Next, immunofluorescence and immunohistochemistry assays revealed that siRP11-79H23.3 group cells revealed much lower PTEN expression as well as a stronger positive signal of p-PI3K and p-Akt expression in tumor tissue of nude mice, whereas higher PTEN expression as well as weaker brown immunostain and fluorescence intensity of p-PI3K and p-Akt were seen in tumor xenograft tissue of RP11-79H23.3 cells group (Figure 5D–G).

### 2.6. MiR-107 Directly Targets RP11-79H23.3/PTEN in BC

**Figure 6:**
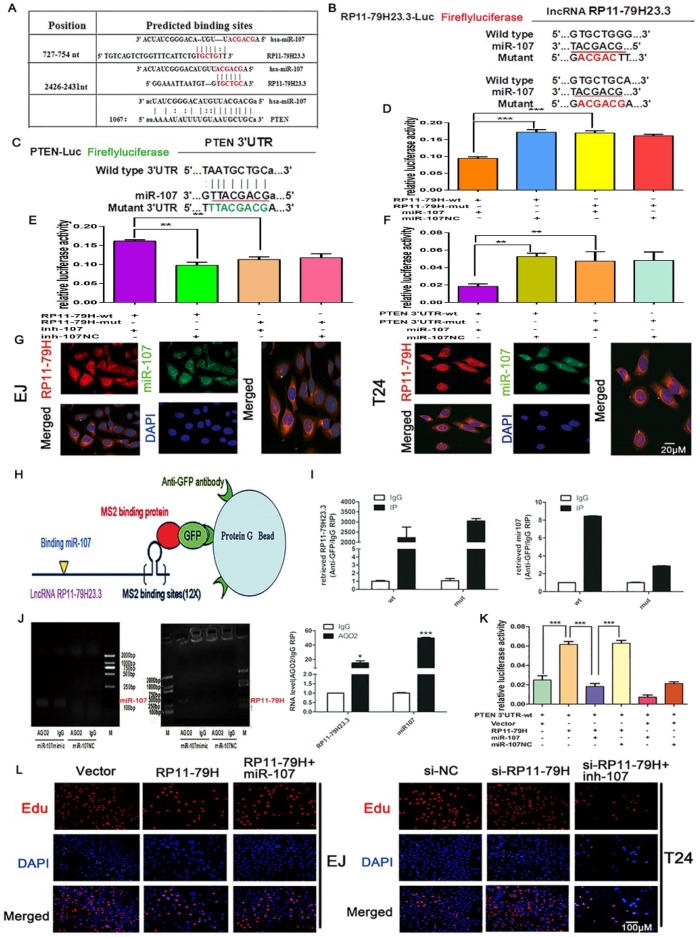

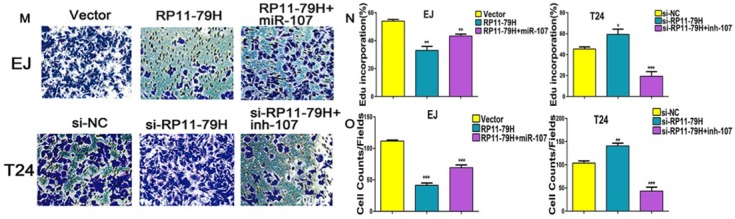
RP11-79H23.3 and PTEN could competitively bind to miR-107. (**A**) The miR-107 binding sites on RP11-79H23.3 and PTEN were predicted by miRcode and TargetScan, respectively. (**B**) Schematic of RP11-79H23.3 wild-type (wt) and mutant (mut) luciferase reporter vector is shown. (**C**) Schematic of miR107 and the putative binding sequence in the 3′-UTR sequence of PTEN as well as the mutant sequence of PTEN 3′-UTR for luciferase assays is shown. (**D**) The relative luciferase activities of EJ cells were tested after co-transfection with pmirGLO-RP11-79H23.3-wt or pmirGLO-RP11-79H23.3-mut and miR-107 mimics or miR-NC. (**E**) The relative luciferase activities were analyzed in EJ cells co-transfected with inh-107 or inh-NC and luciferase reporter vectors pmirGLO-RP11-79H23.3-wt or pmirGLO-RP11-79H23.3-mut. (**F**) The relative luciferase activities were analyzed in EJ cells co-transfected with miR-107 mimics or miR-NC and luciferase reporter vectors pmirGLO-PTEN 3′UTR-wt or pmirGLO- PTEN3′UTR-mut. (**G**) The subcellular localization for RP11-79H23.3 and miR-107 was detected in BC cells with FISH (scale bar, 20 μm). (**H**) The schematic diagram of GFP-MS2-RIP is shown. (**I**) GFP-MS2-RIP followed by qRT-PCR to detect miR-107 and endogenously associated RP11-79H23.3 in EJ cells. (**J**) Anti-AGO2 RIP was executed in EJ cells transiently transfected with miR-107 mimics, followed by RT-PCR and qRT-PCR to detect miR-107 and RP11-79H23.3 associated with AGO2. (**K**) The relative luciferase activities were analyzed in EJ cells co-transfected with luciferase reporter vectors pmirGLO-PTEN 3′UTR-wt and pIRES2-RP11-79H23.3 or vector and/or miR-107 mimics or miR-NC. (**L**,**N**) EdU assay showed that ectopic expression of miR-107 abolished the decreased proliferation by RP11-79H23.3 overexpression in EJ cells, whereas miR-107 inhibitor reversed the increased proliferation by RP11-79H23.3 knockdown in T24 cells (scale bar, 100 μm). (**M**,**O**) Transwell assay indicated that overexpression of miR-107 relieved the reduced invasion by upregulation of RP11-79H23.3 in EJ cells, whereas co-transfected miR-107 inhibitor abated the increased invasion by siRP11-79H23.3 in T24 cells (scale bar, 100 μm). Each experiment was independently repeated at least three times. Data is shown as mean ± SD, * *p* < 0.05, ** *p* < 0.01, *** *p* < 0.001.

To clarify the molecular mechanism underlying RP11-79H23.3, firstly, bioinformatics analysis was executed by miRcode and TargetScan. The data showed that both RP11-79H23.3 and PTEN contain conserved target sites of miR-107 (Figure 6A). Next, dual-luciferase reporter assays were performed to detect the binding between RP11-79H23.3 and miR-107. RP11-79H23.3/PTEN wild and mutant dual-luciferase plasmids were constructed. The 3′UTR of RP11-79H23.3 and PTEN were subcloned to the pmirGLO dual-luciferase reporter vectors (Figure 6B,C). The data showed that transfection of miR-107 mimics could obviously reduce the activity of a luciferase reporter carrying the 3′-UTR of RP11-79H23.3 of WT but not mutant of 3′-UTR, while transfection of the miR-107 inhibitor (inh-107) could remarkably increase the luciferase activity of WT reporters but not the mutant one compared with controls (Figure 6D,E). Moreover, the luciferase reporter activity was significantly decreased in EJ cells co-transfected with miR-107 mimic and pmirGLO-PTEN-WT vector but not the mutant one compared with other controls (Figure 6F). In addition, we investigated the subcellular localization of RP11-79H23.3 and miR-107 in BC cells by FISH. The results indicated that RP11-79H23.3 (red) and miR-107 (green) mainly distributed in cytoplasm, the orange region showed that RP11-79H23.3 and miR-107 are colocalized in the EJ and T24 cells (Figure 6G). Subsequently, to further confirm the endogenous binding between RP11-79H23.3 and miR-107 at the cellular level, we performed RNA immunoprecipitation (RIP) to pull down miRNAs connected with RP11-79H23.3 and confirmed this with qRT-PCR. The data demonstrated that the RIP of RP11-79H23.3 was remarkably enriched for miR-107 in EJ cells compared with MS2 the empty vector and RP11-79H23.3 mutations with miR-107 targeting site vector groups (Figure 6H,I). The miRNAs negatively regulate gene expression in an AGO2-dependent manner. Therefore, we further executed anti-AGO2 RIP in EJ cells transfected with miR-107. Endogenous RP11-79H23.3 pull-down by AGO2 was specifically enriched in the overexpressing miR-107 cells (Figure 6J). Furthermore, we co-transfected the luciferase reporter of pmirGLO-PTEN 3′UTR-wt with pIRES2-RP11-79H23.3, and the data revealed that the luciferase activity was significantly enhanced compared with the control group of co-transfection with WT luciferase reporter and vector, suggesting that upregulation of RP11-79H23.3 increased luciferase activity of pmirGLO-PTEN 3′UTR-wt reporter by competitively binding endogenous miR-107. However, this effect could be obviously abrogated by overexpression of miR-107 (Figure 6K). More importantly, ectopic expression of miR-107 significantly counteracted the proliferation and invasion-suppressing effects of RP11-79H23.3 overexpression in EJ cells, whereas miR-107 inhibitor attenuated the promoting effects mediated by RP11-79H23.3 knockdown in T24 cells by EdU and transwell assays (Figure 6L–O). Collectively, these data demonstrated that miR-107 could directly target RP11-79H23.3 and bind to PTEN in BC cells.

### 2.7. RP11-79H23.3 Regulates the Expressions of PTEN and PI3K/AKT Signaling Pathway Molecules In Vitro

**Figure 7:**
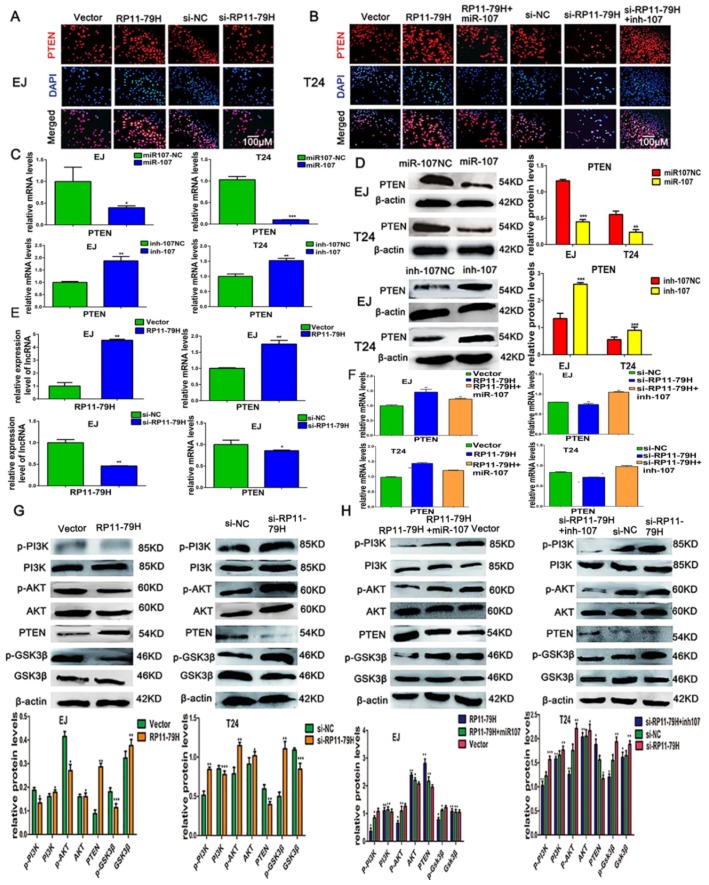
RP11-79H23.3 and miR-107 impact the expressions of the target gene PTEN and PI3K/AKT signaling pathway molecules in vitro. (**A**,**B**) Immunofluorescent assays were used to detect the expressions of PTEN in EJ and T24 cells transfected with pIRES2-RP11-79H23.3 or siRP11-79H23.3 or pIRES2-RP11-79H23.3 and miR-107 mimics or siRP11-79H23.3 and inh-107 (scale bar, 100 μm). (**C**) The mRNA levels of PTEN were determined by qRT-PCR in EJ and T24 cells after being transfected with miR-107 mimics or inhibitors. (**D**) The protein levels of PTEN were determined in EJ and T24 cells transfected with miR-107 mimics or inhibitors by Western blotting. (**E**) The expressions of RP11-79H23.3 and PTEN were detected in cells transfected with pIRES2-RP11-79H23.3 and si RP11-79H23.3 by qRT-PCR. (**F**) qRT-PCR assays were performed to detect the mRNA levels of PTEN in EJ and T24 cells transfected with pIRES2-RP11-79H23.3 or siRP11-79H23.3 or pIRES2-RP11-79H23.3 and miR-107 mimics or siRP11-79H23.3 and inh-107. (**G**,**H**) The expressions of PTEN and PI3K/AKT signaling pathway molecules were assayed in BC cells transfected with the indicated plasmids or siRNAs or pIRES2-RP11-79H23.3 and miR-107 mimics or siRP11-79H23.3 and inh-107 by Western blotting. Each experiment was independently repeated at least three times. Data are shown as mean ± SD, * *p* < 0.05, ** *p* < 0.01, *** *p* < 0.001.

**Figure 8:**
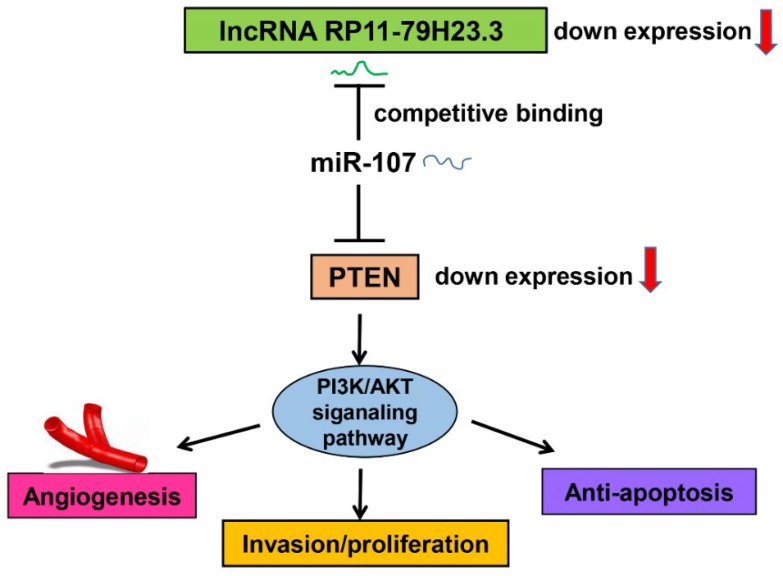
Schematic diagram of the proposed mechanism showing how RP11-79H23.3 as a ceRNA regulates the PTEN/PI3K/AKT pathway in the pathogenesis and development of BC.

To further elucidate the underlying mechanism of RP11-79H23.3 in BC, we determined whether RP11-79H23.3 could regulate the target gene PTEN and the PI3K/AKT signaling pathway. The results of an immunofluorescence assay for PTEN indicated that upregulation of RP11-79H23.3 markedly enhanced the expression of PTEN and the increased impact could be attenuated by ectopically expressing miR-107, whereas downregulation of RP11-79H23.3 observably decreased the PTEN level and the reduced role could be reversed by miR-107 inhibitor (Figure 7A,B). Furthermore, we found that ectopic expression of miR-107 significantly inhibited the level of PTEN, whereas knockdown of miR-107 obviously increased expression of PTEN by qRT-PCR and Western blot assays in BC cells (Figure 7C,D). Additionally, our data showed that the expression of PTEN was significantly upregulated in BC cells transfected with the pIRES2-RP11-79H23.3, whereas siRP11-79H23.3 could remarkably reduce the expression of PTEN with qRT-PCR compared with control groups (Figure 7E). The co-transfection of RP11-79H23.3 and miR-107 could diminish the expression of PTEN compared with RP11-79H23.3 alone in BC cells, while co-transfection of siRP11-79H23.3 and miR-107 inhibitor could abolish the miR-107 inhibitor-induced upregulation of PTEN in BC cells with qRT-PCR (Figure 7F). Subsequently, the Western blot assay showed that upregulation of RP11-79H23.3 significantly decreased the levels of p-PI3K, p-AKT, and p-GSK3β in BC cells as well as increased the expression of PTEN, whereas si-RP11-79H23.3 led to the activation of the PI3K/AKT pathway. Consistently, the co-transfection of miR-107 and RP11-79H23.3 attenuated the expression of PTEN as well as enhanced the phosphorylation level of PI3K, AKT, and GSK3β compared with RP11-79H23.3 alone in EJ cells, while si-RP11-79H23.3 abolished the effects caused by inhibition of miR-107 in T24 cells (Figure 7G,H). Collectively, these data demonstrated that RP11-79H23.3 might function as a ceRNA to regulate the PTEN/PI3K/AKT pathway in the pathogenesis and development of BC (Figure 8).

## 3. Discussion

It has been documented that lncRNAs play important roles in cancer development and progression, but most of them have not yet been studied in functional and mechanistic detail. In the present study, we identified a lncRNA RP11-79H23.3 which was downregulated in BC based on microarray gene-expression profile analysis. RT–qPCR analysis showed that RP11-79H23.3 levels were significantly downregulated in BC tissues and cells. We first demonstrate that RP11-79H23.3 could function as a ceRNA to regulate PTEN expression by sponging miR-107. Our data suggest that RP11-79H23.3 might play a role as a tumor suppressor in the tumorigenesis and progression of BC.

The tumor suppressor PTEN dephosphorylates the D3 position of phosphatidylinositol-3,4,5 triphosphate (PIP3) to negatively control PI3K activity and thus inhibits a panel of cellular responses mediated by the PI3K/Akt pathway including cell growth, mobility, and invasion [20]. Tsuruta et al. found that PTEN expression was significantly reduced in bladder cancer patients, and this decrease in PTEN correlated with disease stage and grade. Thus, PTEN deficiency may contribute to initiation and progression of bladder cancer [21]. Recently, some miRNAs have been demonstrated to promote tumorigenesis and metastasis by downregulating PTEN expression. MiR-130b could target PTEN to mediate drug resistance and proliferation of breast cancer cells via the PI3K/Akt signaling pathway [22]. MiR-106b and miR-93 were reported to regulate cell progression by suppression of PTEN via PI3K/Akt pathway in breast cancer [23]. MiR-21 promoted proliferation and invasion of triple-negative breast cancer cells through targeting PTEN [24]. However, whether miR-107 can target PTEN in BC has not yet been reported. On the basis of bioinformatics prediction and luciferase reporter assay, we further showed miR-107 mimics significantly reduced the PTEN level, whereas miR-107 inhibitor observably enhanced PTEN expression by qRT-PCR and Western blot assays. We provided the evidence that PTEN was a direct target of miR-107 in BC.

In recent years, a ceRNA hypothesis has been proposed that RNA transcripts of coding RNAs and noncoding RNAs can communicate with each other to regulate gene expression by competing for binding to shared miRNAs [11]. It has been reported that the abnormally expressed lncRNAs could act as ceRNAs for miRNAs to regulate the expression of miRNA target genes and disrupt the equilibrium of ceRNAs and miRNAs in tumor development. Long noncoding RNA MEG3 could function as a competing endogenous RNA for miR-181a to regulate carcinogenesis and progression of gastric cancer [25]. LncRNA HOTAIR (HOX transcript antisense RNA) regulated HIF-1α (hypoxia inducible factor 1 subunit alpha)/AXL (AXL receptor tyrosine kinase) signaling and promoted tumorigenesis through acting as a ceRNA of miR-217 in renal cell carcinoma [26]. Long noncoding RNA HNF1A-AS1 (HNF1A antisense RNA1) acted as a ceRNA for miR-30b-5p to promote proliferation and suppress apoptosis of bladder cancer cells through upregulating Bcl-2 [27]. Here, we report a lncRNA, lnc-RP11-79H23.3, that might function as a tumor suppressor in human BC. The data showed that the expression of RP11-79H23.3 was positively correlated with the level of PTEN. The luciferase reporter assays found that RP11-79H23.3 and PTEN could compete for binding to miR-107. In addition, RIP assay verified that RP11-79H23.3 could directly bind to miR-107 in an AGO2-dependent manner. More importantly, RP11-79H23.3 might regulate PTEN expression by antagonizing miR-107. Together, these data confirm that RP11-79H23.3 might function as a competing endogenous RNA for miR-107 in the development of BC.

In conclusion, we found that RP11-79H23.3 was downregulated in bladder cancer and could liberate miR-107 via its function as a ceRNA to suppress PTEN expression and activate the PI3K/Akt signaling pathway, which consequently contributes to the pathogenesis and progression of BC. Taken together, our findings suggest that the RP11-79H23.3 could play a potential tumor suppressor role in the progression and development of BC. The RP11-79H23.3/miR-107/PTEN axis might serve as a novel clinical marker and therapeutic target for BC.

## 4. Materials and Methods

### 4.1. Patient and Tissue Samples

Bladder cancer tissues and pair-matched adjacent tissues used in this paper were obtained from the First Affiliated Hospital of Chongqing Medical University and the Affiliated Hospital of Southwest Medical University during 2010–2015 in accordance with the Helsinki Declaration. None of the patients had undergone radiation treatment or chemotherapy before surgery. Informed consent was obtained from these patients. Tumors were classified according to the tumor–node–metastasis (TNM, 2010) system of classification. The clinicopathological characteristics of the BC patients are summarized in Table 1. This study was approved by the Ethics Committee of Chongqing Medical University.

### 4.2. Cell Lines, Plasmid Construction, and Transfection

Human normal bladder epithelial cell line (SV-HUC-1) and bladder cancer cell lines (EJ, T24, BIU87) were obtained from the American Type Culture Collection (Manassas, VA, USA). Cells were maintained in a humidified incubator at 37 °C in an atmosphere of 5% CO_2_. EJ, T24, BIU87 and SV-HUC-1 cells were maintained in RPMI-1640 and DMEM/F12K medium, respectively.

Full-length RP11-79H23.3 from 293 cells was amplified by PCR and then cloned into pIRES2-EGFP vector (Shanghai, China). The sequences of RP11-79H23.3 3′-UTR and the sequence of PTEN 3′-UTR containing putative miR-107 binding sites and the corresponding mutant (Mut) 3′-UTR sequences were amplified (F-RP11-79H23.3-Wt: GCGGCTCGAGGTCGTGGACTAATGAAACA, R-RP11-79H23.3-Wt: AATGCGGCCGCTACCAATGACATGAAAGGG, F-RP11-79H23.39-Mut1: TCATTCTGACGACTTAACTTCTACATGGTGC, R-RP11-79H23.39-Mut1: GAAGTTAAGTCGTCAGAATGAAACCAGACTG, F-RP11-79H23.39-Mut2: TTAATGTGACGACGATTATAACCTAGGTTATT, R-RP11-79H23.39-Mut2: GTTATAATCGTCGTCACATTAATTTCCAAATC, F-PTEN-3′UTR-Wt: GCGGCTCGAGACCAACTGAAGTGGCTAA, R-PTEN-3′UTR-Mut: ACGATCCTGTCGTCCTATTATGATTGAAAACT, F-PTEN-3′UTR-Mut: TCATAATAGGACGACAGGATGCTTCATGTGCTG, R-PTEN-3′UTR-Wt: AATGCGGCCGCAAACTCTATAAATGCTGC) and then cloned into the pmiR-RB-Report™ vector (RIBOBIO, Guangzhou, China). si-RP11-79H23.3 (sense I: 5′-GCTTCAACTCTGTGAAATA-3′, sense II: 5′-CCTATTTCTTACCATCCTT-3′) was synthesized by RIBOBIO, miR-107 mimics, miR-107 inhibitor, and negative control, which were from GenePharma (Shanghai, China). The transfections were executed with a Lipofectamine 2000 (Invitrogen, Carlsbad, CA, USA) according to the manufacturer’s guide.

### 4.3. LncRNA and mRNA Microarray

Briefly, as previously reported (Huang et al., 2016 [15]), four pairs of human BC and the adjacent normal tissues were applied for microarray assay to determine differentially expressed lncRNAs and mRNAs by the Arraystar Human lncRNA Array v2.0 (KangChen Bio-tech, Shanghai, China). The transcript of the significant differential expression was reserved by fold change ≥2.0, *p* < 0.05, and FDR < 0.05. Hierarchical clustering was implemented to produce an outline of expression patterns according to the expression value of all the transcripts with or without significant differences. Microarray data were available through Gene Expression Omnibus (GEO) with accession code GSE89006.

### 4.4. qRT-PCR Assay

Total RNA was extracted from the frozen tissues and cultured cells with TRIzol (TAKARA, Dalian, China). The quantity and quality of RNA were detected by Nano Drop 2000 spectrophotometer (Nano Drop Thermo, Wilmington, DE, USA), and the integrity of RNA was evaluated by agarose gel electrophoresis. cDNA was generated with a reverse-transcription kit (Takara, Dalian, China) according to manufacturer’s protocol. The qRT-PCR analysis was implemented by the ABI 7900HT Sequence Detection Machine (Bio-Rad, Hercules, CA, USA) using SYBR Green chemistry. U6 or GAPDH was used as an endogenous control. The specific primers sequences were as follows: RP11-79H23.3, 5′-TGGCCTCAGTTAGGACTGCT-3′ and 5′-CTGCTTCCGCTCTCTTTCTC-3′; PTEN, 5′-GCTATGGGATTTCCTGCAGAA-3′ and 5′-GGCGGTGTCATAATGTCTTTCA-3′; GAPDH, 5′-GAAGGTGAAGGTCGGAGTC-3′ and 5′-GAAGATGGTGATGGGATTTC-3′. PCR was executed with an initial denaturation step at 95 °C for 5 min, followed by amplification with 40 cycles at 95 °C for 10 s and 60 °C for 35 s, the melt curve step at 60 °C to 95 °C, and the increment at 0.5 °C for 5 s. The genes were amplified in separate wells in triplicate. The relative expressions levels were calculated with the (2^−∆∆*C*t^) method.

### 4.5. Cell Proliferation, Viability, and Colony Assays

The cell proliferation ability was determined by CCK8, EdU and colony formation assays. For CCK-8 assay, Cell Counting Kit-8 was bought from DingGuo (Beijing, China), and cells were seeded into 96-well plates (5000 cells/well) with complete growth medium. After incubation for 24, 48, 72, and 96 h, respectively, 10 μL CCK-8 was added into each well, and then the cells were cultured for an additional 2 h. The absorbance value was tested by a plate reader at 450 nm (Bio-Rad, Hercules, CA, USA). 1 × 10^5^ cells were put into 24-well plates for EdU incorporation with an EdU detection kit (Ribobio, Guangzhou, China). The percentage of EdU-positive cells was quantified from four random fields per well. For colony formation assays, 2.5 × 10^2^ cells, were plated onto six-well plates and incubated for 14 days, fixed with 4% paraform, then stained with 0.1% crystal violet, and the number of colonies in four random fields were counted under an inverted microscope. The experiments were performed in triplicate.

### 4.6. Wound Healing, Cell Invasion Assay, and Cytoskeleton

When cells grew in six-well plates to 80–90% confluence, scratches were made with a 200 μL tip, they were washed with PBS (Phosphate Buffered Saline), and then the cells were further incubated in FBS (fetal bovine serum)-free medium. The width of wound closure (original width at 0 h–width after cell migration at 24 h) was counted in the same wound point with five replicates. The cell invasion assays were performed using 24-well Transwell chambers with a pore size of 8 μm (BD BioCoat, Bedford, MA, USA). 5 × 10^5^ cells in serum-free RPMI1640 were added to the upper chamber coated with matrigel (BD Biosciences, Franklin Lakes, NJ, USA), and the bottom chamber contained 500 µL of culture medium with 10% FBS. After 24 h, noninvading cells on the upper chamber were rubbed away with a cotton bud and invading cells in the lower chamber were stained by crystal violet, photographed, and counted with an Olympus multifunction microscope (Tokyo, Japan). Assays were repeated three times. The cells were seeded into 24-well plates after 48 h, rinsed with PBS, fixed in 4% paraformaldehyde, permeabilized by 0.3% Triton X-100 at 37 °C for 6 min, then rinsed three times with PBS, and blocked with 3% BSA (bovine serum albumin) at 37 °C for 30 min. The cells were incubated with Paxillin antibody (1:200 dilution) overnight at 4 °C, then incubated with IgG-TRITC for 1 h, then incubated with 1% FITC-phalloidin (Sigma Chemical Corp., St. Louis, MO, USA) at 37 °C for 30 min. The cells were stained by DAPI (4′,6-diamidino-2-phenylindole, SouthernBiotech, Birmingham, AL, USA) for 10 min, sealed with antifluorescent solution, and were then were observed under a confocal laser scanning microscope (Leica, Wetzlar, Germany).

### 4.7. Cell Apoptosis and Cell Cycle Assay

The cells were fixed with 4% paraform for 20 min, then dyed with Hoechst 33342 for 15 min and observed under a Leica TCS-SP2 Laser Confocal Microscopy. Apoptosis was assessed by TUNEL assay using an apoptosis detection kit (Keygen Biotech, Nanjing, China). The FITC-labeled apoptotic cells were photographed under a fluorescent microscope. The cells were harvested and apoptosis was determined with flow cytometry (Becon Dickinson FACSCalibur, New York, NY, USA) after annexin V-FITC/PI double staining. 2 × 10^6^ cells were fixed for 12 h in 70% ethanol, then stained with PI. The cell cycle was observed with flow cytometry (Becon Dickinson FACSCalibur, New York, NY, USA).

### 4.8. Fluorescence In Situ Hybridization (FISH)

The subcellular localizations of LncRNA RP11-79H23.3 and miR-107 were examined by FISH kit (Roche Applied Science, Penzberg, Germany). Cells grew to 70% confluence, were fixed with 4% formaldehyde for 15 min, then permeabilized with 0.5% TritonX-100 for 15 min and rinsed with PBS. Cells were incubated in a mixture of RP11-79H23.3 probes labeled with Cy3 at 37 °C overnight and washed with prewarmed 2× saline-sodium citrate (SSC). The FITC-labeled miR-107 probes were incubated in prehybridization buffer (1:100) at 88 °C for 5 min and at 4 °C for 3 min with the PCR instrument (Bio-Rad, Hercules, CA, USA). Next, the FITC-labeled miR-107 probes were added into cells at 37 °C overnight, washed with 2× SSC, and stained by DAPI. Observations were undertaken with a Leica TCS-SP2 Laser Scanning Confocal Microscope.

### 4.9. Dual-Luciferase Reporter Assay

Pmir-RB-RP11-79H23.3(RP11-79H-wt), pmir-RB-RP11-79H23.3-mutant (RP11-79H-mut), pmir-RB-PTEN (PTEN 3′-UTR-wt), or pmir-RB-PTEN-mutant (PTEN 3′-UTR-mut) was co-transfected using Lipofectamine 2000 with miR-107 mimic or NC-mimic and miR-107 inhibitor or NC-inhibitor or pIRES2-RP11-79H23.3 into EJ cells. According to the manufacturer’s protocol, luciferase activities were determined using the dual-luciferase assay kit (Promega, Madison, WI, USA) after 48 h of transfection.

### 4.10. RIP (RNA Immunoprecipitation) Assay

RIP is an antibody-based technique used to map in vivo RNA-protein interactions. The RNA binding protein (RBP) of interest is immunoprecipitated together with its associated RNA for identification of bound transcripts (mRNAs, noncoding RNAs, or viral RNAs). Transcripts are detected by real-time PCR. RIP detection was implemented according to the manufacturer’s guide with the Magna RNA binding protein immunoprecipitation kit (Millipore, Billerica, MA, USA). EJ cells were transfected with miR-107 mimics and miR-107 NC, respectively. Then, after cells were lysed by RIP lysis buffer, whole cell lysate was incubated in the buffer containing anti-AGO2 antibody coupled to magnetic beads (Millipore). RNA was separated and deposited by immunoprecipitation, then the products were purified and analyzed by qRT-PCR. In addition, EJ cells were co-transfected with pcDNA3.1-MS2, pcDNA3.1-MS2-RB-RP11-79H23.3, and pcDNA3.1-MS2-RB-RP11-79H23.3-mut (no miR-107 binding site), respectively, and pMS2-GFP (Addgene plasmids). After 48 h, RIP experiments were executed using an anti-GFP antibody (Abcam, Burlingame, CA, USA) with IgG antibody as a negative antibody. The products were purified and enriched to detect the target miRNAs by qRT-PCR.

### 4.11. Western Blot Analysis

Cell protein lysates were extracted from cells using 1× sodium dodecyl sulfate buffer and quantified by Bicin Choninic Acid (BCA) protein assay kit (Thermo, Waltham, MA, USA). On a 10% SDS-PAGE (Sodium doecylsulfate-polyacrylamide gel electrophoresis), 30 μg of protein samples per lane were separated and then electransferred to a PVDF (polyvinylidene fluoride) membrane. The samples were blocked in 5% nonfat milk powder and then incubated with antibodies against PTEN (Abcam, Burlingame, CA, USA), AKT, p-AKT, PI3K, p-PI3K,GSK3β, p-GSK3β, Bax, Bcl-2, Caspase-3 cleaved (Cell Signaling Technology, Beverly, MA, USA), and β-actin (Proteintech, Rosemont, IL, USA) at 4 °C overnight. Blots were incubated with goat anti-rabbit secondary antibodies (Bioss, Beijing, China) for 2 h at room temperature. β-actin was used as an internal control. The bands were visualized by an ECL chemiluminescent detection system (Thermo Scientific, New York, NY, USA).

### 4.12. Immunohistochemistry (IHC) and Immunofluorescence (IF)

Cells were cultured on the slides in a 24-well plate and fixed. The frozen and paraffin section were obtained from nude mouse tumor tissues and human tissues for IF and IHC, respectively. For IHC assay, after dewaxing, rehydration, and antigen retrieval, the slides were then incubated with antibodies specific for PTEN and PI3K/AKT signaling pathway molecules (Abcam) and secondary HRP labelled goat anti-rabbit, stained with DAB, then counterstained with hematoxylin. For IF assays, the cells and frozen section were incubated with indicated primary antibodies overnight at 4 °C. Then, samples were incubated with fluorescein Alexa-Fluor 488- or 594-conjugated secondary antibodies. The slides were dyed by DAPI, then observed under a confocal laser microscope and Olympus multifunction microscope.

### 4.13. Tumor Xenograft Model

BALB/c nude mice (4–6 weeks old, female) were purchased from Beijing HFK Bioscience Co., Ltd. (Beijing, China). The stably transfected EJ cells (2 × 10^6^) with indicated vector or 3′-cholesterol and 2′-OMe-conjugated siRNA and NC random fragments (RIBOBIO) were subcutaneously injected into BALB/c mice, respectively. The volume of tumor was measured per week. The mice were sacrificed after 35 days, and tumor xenografts and lungs were harvested and subjected to pathologic, immunohistochemical, and immunofluorescent examination. The microvessels were counted from six different fields in the tumor HE sections under microscope. Metastatic nodules of the lung were recorded under the microscope. All animal care and experiment procedures were implemented according to the guidelines of the National Institutes of Health. The study was conducted in accordance with the Declaration of Helsinki, and the protocol was approved by the Ethics Committee of Chongqing Medical University (Date: 16 February 2016, Identification code: 2016021606).

### 4.14. Statistical Analysis

Statistical analyses were executed by Student’s *t*-test and One-way ANOVA using GraphPad Prism 6.0 and SPSS 19.0 statistical software. The significance of differences between groups was assessed by Student’s *t* test and Fisher’s exact test. The data were expressed as mean ± SD. *p* < 0.05 was considered as statistical significance.

## Figures and Tables

**Table 1:** Correlation between the RP11-79H23.3 expression and the clinicopathologic features of bladder cancer.

Characteristic		RP11-79H23.3		*p* Value
		Low	High	
All patients		15	15	
Gender	Male	12	9	0.427
	Female	3	6	
Age at diagnosis	<55 years	6	10	0.272
	≥55 years	9	5	
Pathologic grade	Low grade	5	8	0.462
	High grade	10	7	
T	<T2	3	10	0.025
	≥T2	12	5	
N	N0	4	11	0.027
	N1/2	11	4	

Statistically significant *p* < 0.05.

## References

[1] Torre L.A., Bray F., Siegel R.L., Ferlay J., Lortet-Tieulent J., Jemal A. (2015). Global cancer statistics, 2012. CA Cancer J. Clin..

[2] Chen H., Lin Y.W., Mao Y.Q., Wu J., Liu Y.F., Zheng X.Y., Xie L.P. (2012). MicroRNA-449a acts as a tumor suppressor in human bladder cancer through the regulation of pocket proteins. Cancer Lett..

[3] Chen J., Wang L., Tang Y., Gong G., Liu L., Chen M., Chen Z., Cui Y., Li C., Cheng X. (2016). Maspin enhances cisplatin chemosensitivity in bladder cancer T24 and 5637 cells and correlates with prognosis of muscle-invasive bladder cancer patients receiving cisplatin based neoadjuvant chemotherapy. J. Exp. Clin. Cancer Res..

[4] Schaukowitch K., Kim T.K. (2014). Emerging epigenetic mechanisms of long non-coding RNAs. Neuroscience.

[5] Wang C., Wang L., Ding Y., Lu X., Zhang G., Yang J., Zheng H., Wang H., Jiang Y., Xu L. (2017). LncRNA Structural Characteristics in Epigenetic Regulation. Int. J. Mol. Sci..

[6] Fatima F., Nawaz M. (2017). Vesiculated Long Non-Coding RNAs: Offshore Packages Deciphering Trans-Regulation between Cells, Cancer Progression and Resistance to Therapies. Noncoding RNA.

[7] Zhan X., Dong C., Liu G., Li Y., Liu L. (2017). Panel of seven long noncoding RNA as a candidate prognostic biomarker for ovarian cancer. OncoTargets Ther..

[8] Fatima R., Akhade V.S., Pal D., Rao S.M. (2015). Long noncoding RNAs in development and cancer: Potential biomarkers and therapeutic targets. Mol. Cell. Ther..

[9] Hayes C.N., Chayama K. (2016). MicroRNAs as Biomarkers for Liver Disease and Hepatocellular Carcinoma. Int. J. Mol. Sci..

[10] Feng B., Zhang K., Wang R., Chen L. (2015). Non-small-cell lung cancer and miRNAs: Novel biomarkers and promising tools for treatment. Clin. Sci..

[11] Salmena L., Poliseno L., Tay Y., Kats L., Pandolfi P.P. (2011). A ceRNA hypothesis: The Rosetta stone of a hidden RNA language?. Cell.

[12] Zou C.D., Zhao W.M., Wang X.N., Li Q., Huang H., Cheng W.P., Jin J.F., Zhang H., Wu M.J., Tai S. (2016). MicroRNA-107: A novel promoter of tumor progression that targets the CPEB3/EGFR axis in human hepatocellular carcinoma. Oncotarget.

[13] Teng R., Hu Y., Zhou J., Seifer B., Chen Y., Shen J., Wang L. (2015). Overexpression of Lin28 Decreases the Chemosensitivity of Gastric Cancer Cells to Oxaliplatin, Paclitaxel, Doxorubicin, and Fluorouracil in Part via microRNA-107. PLoS ONE.

[14] Molina-Pinelo S., Carnero A., Rivera F., Estevez-Garcia P., Bozada J.M., Limon M.L., Benavent M., Gomez J., Pastor M.D., Chaves M. (2014). MiR-107 and miR-99a-3p predict chemotherapy response in patients with advanced colorectal cancer. BMC Cancer.

[15] Huang M., Zhong Z., Lv M., Shu J., Tian Q., Chen J. (2016). Comprehensive analysis of differentially expressed profiles of lncRNAs and circRNAs with associated co-expression and ceRNA networks in bladder carcinoma. Oncotarget.

[16] Wang X., Huang H., Young K.H. (2015). The PTEN tumor suppressor gene and its role in lymphoma pathogenesis. Aging.

[17] Calderaro J., Rebouissou S., de Koning L., Masmoudi A., Hérault A., Dubois T., Maille P., Soyeux P., Sibony M., de la Taille A. (2014). PI3K/AKT pathway activation in bladder carcinogenesis. Int. J. Cancer.

[18] Yang X., Cheng Y., Li P., Tao J., Deng X., Zhang X., Gu M., Lu Q., Yin C. (2015). A lentiviral sponge for miRNA-21 diminishes aerobic glycolysis in bladder cancer T24 cells via the PTEN/PI3K/AKT/mTOR axis. Tumor Biol..

[19] Egawa H., Jingushi K., Hirono T., Ueda Y., Kitae K., Nakata W., Fujita K., Uemura M., Nonomura N., Tsujikawa K. (2016). The miR-130 family promotes cell migration and invasion in bladder cancer through FAK and Akt phosphorylation by regulating PTEN. Sci. Rep..

[20] Ma Q., Wu X., Wu J., Wu H., Xiao Y., Wang L., Liang Z., Liu T. (2017). PDZ-containing 1 acts as a suppressor of pancreatic cancer by regulating PTEN phosphorylation. Oncotarget.

[21] Tsuruta H., Kishimoto H., Sasaki T., Horie Y., Natsui M., Shibata Y., Hamada K., Yajima N., Kawahara K., Sasaki M. (2006). Hyperplasia and carcinomas in Pten-deficient mice and reduced PTEN protein inhuman bladder cancer patients. Cancer Res..

[22] Miao Y., Zheng W., Li N., Su Z., Zhao L., Zhou H., Jia L. (2017). MicroRNA-130b targets PTEN to mediate drug resistance and proliferation of breast cancer cells via the PI3K/Akt signaling pathway. Sci. Rep..

[23] Li N., Miao Y., Shan Y., Liu B., Li Y., Zhao L., Jia L. (2017). MiR-106b and miR-93 regulate cell progression by suppression of PTEN via PI3K/Akt pathway in breast cancer. Cell Death Dis..

[24] Fang H., Xie J., Zhang M., Zhao Z., Wan Y., Yao Y. (2017). miRNA-21 promotes proliferation and invasion of triple-negative breast cancer cells through targeting PTEN. Am. J. Transl. Res..

[25] Peng W., Si S., Zhang Q., Li C., Zhao F., Wang F., Yu J., Ma R. (2015). Long non-coding RNA MEG3 functions as competing endogenousRNA to regulate gastric cancer progression. J. Exp. Clin. Cancer Res..

[26] Hong Q., Hong Q., Zheng W., Xiao W.Z., Zhang L., Wu D., Cai G.Y., He J.C., Chen X.M. (2017). LncRNA HOTAIR regulates HIF-1α/AXL signaling through inhibition of miR-217 in renal cell carcinoma. Cell Death Dis..

[27] Zhan Y., Li Y., Guan B., Wang Z., Peng D., Chen Z., He A., He S., Gong Y., Li X. (2017). Long non-coding RNA HNF1A-AS1 promotes proliferation and suppresses apoptosis of bladder cancer cells through upregulating Bcl-2. Oncotarget.

